# Reading and Generalist Genes

**DOI:** 10.1111/j.1751-228X.2007.00018.x

**Published:** 2007-12

**Authors:** Claire M A Haworth, Emma L Meaburn, Nicole Harlaar, Robert Plomin

**Affiliations:** 1 Social, Genetic, and Developmental Psychiatry Centre, Institute of Psychiatry, King’s College London

## Abstract

Twin-study research suggests that many (but not all) of the same genes contribute to genetic influence on diverse learning abilities and disabilities, a hypothesis called *generalist genes*. This generalist genes hypothesis was tested using a set of 10 DNA markers (single nucleotide polymorphisms [SNPs]) found to be associated with early reading ability in a study of 4,258 7-year-old children that screened 100,000 SNPs. Using the same sample, we show that this early reading SNP set also correlates with other aspects of literacy, components of mathematics, and more general cognitive abilities. These results provide support for the generalist genes hypothesis. Although the effect size of the current SNP set is small, such SNP sets could eventually be used to predict genetic risk for learning disabilities as well as to prescribe genetically tailored intervention and prevention programs.

Two decades of research have made it clear that genetics is a large part of the answer to the question of why children differ in their ability to learn in school. Most research uses the classical twin method that compares resemblance for genetically identical twins (identical, monozygotic, MZ) and for twins who are only 50% similar genetically (nonidentical, dizygotic, DZ). Genetic influence on a trait is indicated to the extent that MZ twins are more similar on the trait than DZ twins, reflecting the twofold greater genetic similarity of MZ as compared to DZ twins. Concordance, a statistic used to indicate twin resemblance, indexes the likelihood that one twin will be affected if the other twin is affected. If a disorder were entirely caused by additive genetic factors, the concordances for MZ and DZ twins would be 100% and 50%, respectively. For reading disabilities, MZ and DZ twin concordances are about 85% and 50%, respectively; for language disabilities, 75% and 45%; and for mathematical disabilities, 70% and 50% (Plomin & Kovas, 2005). These results indicate substantial genetic influence on learning disabilities and greater genetic influence than for most other common psychiatric disorders, such as schizophrenia (50% and 20%), depression (45% and 30%), and alcoholism (50% and 35%) (Plomin, DeFries, McClearn, & McGuffin, in press).

Because the case for genetic influence is so strong for learning disabilities, especially for reading (Olson, 2007; Schulte-Körne et al., 2007;), genetic research has gone beyond the rudimentary nature/nurture question in several ways. Two of the most important directions are multivariate genetic analysis and molecular genetic analysis. The present study brings together these two developments in relation to individual differences in reading in the early school years.

## Multivariate genetic analysis: Generalist genes

As reviewed in the inaugural issue of this journal (Plomin, Kovas, & Haworth, 2007), multivariate genetic research points to the surprising finding that many of the same genes affect different learning abilities and disabilities. Multivariate genetic analysis considers not only the variance of traits considered one at a time but also the covariance among traits. It yields a statistic called the *genetic correlation*, which can be roughly interpreted as the likelihood that genes found to be associated with one trait will also be associated with the other trait. In a review of a dozen multivariate genetic studies of learning abilities and disabilities, the average genetic correlation was about .70 between reading and language performance, between reading and mathematics, and between language and mathematics (Plomin & Kovas, 2005). In other words, if genes were found that are associated with reading disability, these multivariate genetic results suggest that there is about a 70% chance that the same genes will also be associated with other learning disabilities such as mathematics disability. Moreover, the general effects of genes appear to extend beyond specific learning abilities such as reading and mathematics to other more general cognitive abilities such as verbal abilities (e.g., vocabulary and word fluency) and nonverbal abilities (e.g., spatial and memory). The average genetic correlation is about .60 between learning abilities and these cognitive abilities (Plomin et al., 2005). It should be emphasized that, because these genetic correlations are not 1.0, these multivariate genetic results also provide evidence for genes that are specific to each learning and cognitive ability. However, what is surprising is the magnitude of these genetic correlations, which implies that genetic overlap among learning and cognitive abilities is substantial. The quantitative genetic research supporting the hypothesis of generalist genes has been described in detail (Plomin & Kovas, 2005).

The concept of generalist genes has far-reaching implications for understanding genetic links between brain, mind, and education (Plomin et al., 2007). It suggests that genetic nosology differs from current diagnoses based on symptoms, blurring distinctions between ostensibly different disabilities such as reading and math disabilities. That is, most of what is going on genetically has broad general effects across disabilities rather than specific effects on just one disability. Spikes in ability profiles may be primarily environmental in origin.

## Molecular genetics

Rather than again reviewing multivariate genetic research that supports the hypothesis of generalist genes, the present article provides an empirical test of the hypothesis. Although multivariate genetic research consistently supports the generalist genes hypothesis, definitive support will come from molecular genetic research. The generalist genes prediction is clear: Most genes associated with reading ability will also be associated with other aspects of literacy, with other learning abilities such as mathematics, and with general cognitive ability. In other words, if we had a set of genes that were found to be associated with reading ability, we could test the generalist genes hypothesis by assessing the extent to which these reading-related genes were also associated with other learning and cognitive abilities. If these reading-related genes were not associated with other learning and cognitive abilities, the generalist genes hypothesis would not be supported.

The problem with testing the generalist genes hypothesis at a molecular genetic level of analysis is that progress toward identifying the responsible genes has been slow. It is generally accepted that this slow progress is largely due to the fact that genetic influence on common disorders such as learning disabilities and complex traits such as learning abilities involves many genes of small effect and as a result are difficult to detect and replicate because very large samples are required (Plomin, 2005). Multiple genes of small effect sizes responsible for genetic influence on common disorders are often called quantitative trait loci (QTLs), because if a trait is influenced by many genes, the genetic effects will be distributed quantitatively as a normal bell-shaped distribution, regardless of whether a diagnostic cutoff is imposed on the quantitative distribution (Plomin, Owen, & McGuffin, 1994). In other words, the QTL perspective suggests that reading disability is not an etiologically distinct disorder but rather the low extreme of the same genetic (and environmental) factors responsible for variation in reading ability throughout the normal distribution.

In contrast, if a single gene were responsible for a disorder, as is the case for thousands of rare disorders, the chromosomal location of the gene can be readily identified using traditional linkage designs that look for coinheritance between the disorder and a genetic marker (a measurable DNA difference, called a *polymorphism*) within large family pedigrees. However, this traditional linkage design cannot detect genes of small effect size. Instead of studying many family members in a few families, QTL linkage designs study a few family members, usually just siblings, in many families, thus increasing the power to detect smaller effect sizes. The first success of QTL linkage was for reading disability in which chromosomal linkages were identified in 1994 (Cardon et al., 1994). Although it has proven difficult to pinpoint the actual genes responsible for these linkages, four candidate genes are currently under scrutiny (Fisher & Francks, 2006; Paracchini, Scerri, & Monaco, 2007; Schulte-Körne et al., 2007;).

Nonetheless, QTL linkage is unable to detect genes of very small effect size. Association designs are much more powerful for detecting small effect sizes because they are based on the population rather than families. That is, whereas linkage designs look for coinheritance patterns in a family, association designs simply compare frequencies of alternative forms of a gene (called alleles) between cases and controls (or low and high groups). For example, allele frequency differences in a dopamine receptor gene (D4 dopamine receptor, *DRD4*) have been reported for hyperactivity; a particular allele shows a frequency of about 25% for children with hyperactivity and about 15% for controls, which yields an odds ratio of 1.9 (Bobb, Castellanos, Addington, & Rapoport, 2005).

Rather than studying a few candidate genes such as dopamine genes that could plausibly be associated with a trait, genome-wide association scans are now possible that can examine hundreds of thousands of DNA markers simultaneously for their association with disorders (Hirschhorn & Daly, 2005). Genome-wide association is made possible by DNA microarrays that are the size of a postage stamp yet can genotype as many as a million of a particular type of DNA marker called a single nucleotide polymorphism (SNP–pronounced “snip”) (Plomin & Schalkwyk, 2007). Several recent genome-wide association studies have reported associations with common disorders including obesity, heart disease, Type 2 diabetes, and bipolar disorder (Diabetes Genetics Initiative of Broad Institute of Harvard and MIT et al., 2007; Wellcome Trust Case Control Consortium, 2007;).

The first association scan of this type reported for reading identified 10 DNA markers associated with reading (Meaburn, Harlaar, Craig, Schalkwyk, & Plomin, in press) in a sample of 5,500 7-year-olds participating in the UK Twins Early Development Study (TEDS; Oliver & Plomin, 2007). The study used a composite reading measure consisting of the Test of Word Reading Efficiency (TOWRE) and a yearlong teacher assessment of reading based on UK National Curriculum (NC) criteria (Harlaar, Dale, & Plomin, 2005). In TEDS, both measures at age 7 are highly heritable (.63 and .74, respectively) and the genetic correlation between them is .79 (Harlaar, Dale, & Plomin, 2005). Using this composite measure of reading performance, the study scanned more than 100,000 SNPs on microarrays for allele frequency differences between the lowest performing children (*N*= 755) and the highest performing children (*N*= 747). SNPs that showed the largest allele frequency differences between the low and the high groups were tested for the QTL hypothesis by assessing associations between genotypes and phenotypes in an independent unselected sample of 4,258 7-year-olds. Ten SNPs were nominally significant in the expected direction across this unselected sample. However, none of these SNP associations accounted for more than 0.5% of the variance of reading ability, despite 99% power to detect them, which is generally the case for genome-wide association scans for complex traits and common disorders. Nonetheless, a cumulative genetic risk index of these 10 SNPs, called a *SNP set*, accounts for about 1% of the variance in reading (Meaburn et al., in press). Even though the effect sizes of individual SNP associations are very small, bigger and better SNP sets may eventually be able to predict significant genetic risk for learning and cognitive abilities and disabilities.

## The present study

The goal of the present study was to use this set of 10 SNPs associated with early reading ability to test the generalist genes hypothesis by examining associations between this reading SNP set and other aspects of literacy, mathematics, and more general cognitive abilities. Multivariate genetic analyses of reading at age 7 in TEDS support the generalist genes hypothesis in relation to other literacy measures (writing and speaking), other learning abilities (mathematics), and general cognitive ability (a composite of verbal and nonverbal abilities). For example, at 7 years, the NC measure of reading yields genetic correlations of .78 with NC writing, .67 with NC speaking, and .78 with NC mathematics (Kovas, Haworth, Dale, & Plomin, 2007); genetic correlations with general cognitive ability were somewhat lower, about .50 (Harlaar, Hayiou-Thomas, & Plomin, 2005).

These multivariate genetic results led us to hypothesize that the 10 SNP sets significantly associated with reading at age 7 will also be significantly associated with these other measures, as predicted by the generalist genes hypothesis. More specifically, the genetic correlations from multivariate genetic analyses suggest that the magnitude of the associations between the reading SNP set and these other literacy and learning ability measures will be almost as strong as the association with reading itself. The lower genetic correlation with general cognitive ability suggests that its association with the reading SNP set will also be lower. Moreover, although we are not aware of multivariate genetic research on this topic, one might expect that the reading SNP set will be more strongly associated with the verbal than the nonverbal component of general cognitive ability.

## Method

The sampling frame for the present study was the TEDS, a large-scale longitudinal study of cognitive and behavioral development in a representative sample of twins born in England and Wales in 1994, 1995, and 1996 (Oliver & Plomin, 2007). The TEDS sample has been shown to be reasonably representative of the general population (Kovas et al., 2007). A total of 1,759 individuals had complete data for all 10 SNPs; however, as described below, we used a missing data option that substituted the population mean for missing SNPs, giving a sample of 4,258 individuals.

### Measures

#### SNP set

**Fig. 1:**
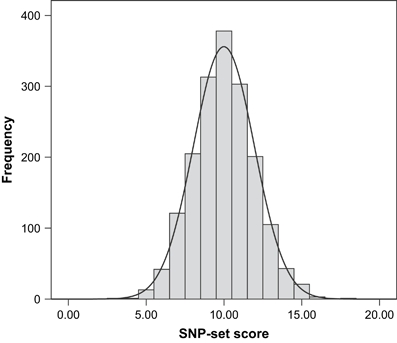
SNP-set distribution. *Note*. This SNP set contains only those individuals who have data for all 10 SNPs. SNP = single nucleotide polymorphism.

The 10 SNPs associated with early reading disability (Meaburn et al., in press) were combined in a SNP set for the current analyses. The additive genotypic values for the 10 SNPs are uncorrelated because the SNPs are not in linkage disequilibrium with each other. This permits the creation of a composite SNP set that aggregates the small effects of each SNP and can be useful in studies that are not sufficiently large to provide the power needed to analyze each SNP separately. Additive genotypic values were coded 0, 1, or 2 for each SNP, with 0 conferring lowest reading ability and 2 conferring highest reading ability. SNP genotypes for the 10 significant associations were summed to produce SNP-set scores from 0 through 20. Only individuals with complete data for all 10 SNPs were included, *N*= 1,759, although analyses were also conducted using a missing data option that substituted the population mean for missing SNPs (*N*= 4,258). The SNP-set scores were normally distributed (see Figure 1). A composite SNP set was used because the individual SNPs accounted for less than 0.5% of the variance for reading. Therefore, by combining these effect sizes in the SNP set, we will eventually be able to predict significant genetic risk. For this reason, we do not present results from the individual SNPs in this article.

#### English

In the following sections, we briefly describe the learning and cognitive measures used in the present analyses. Much more detail about the validity and reliability of these measures is available elsewhere (Kovas et al., 2007). We collected teacher assessments of three domains of English performance: speaking and listening, reading, and writing. These assessments were based on Key Stage 1 of the UK NC, the core academic curriculum developed by the Qualifications and Curriculum Authority (QCA; http://www.qca.org.uk). For the NC teacher assessments, teachers summarize students’ performance throughout the school year in each of these areas using a 5-point scale. This judgment forms the continuing assessment of each child that ultimately leads to the final NC teacher assessment score submitted to the QCA at the end of the school year. In addition, we calculated an English composite score, which was the mean of the three scales.

#### Mathematics

Similar UK NC teacher reports of mathematic performance were also collected based on Key Stage 1 criteria. Teachers provided scores for three domains of mathematics: using and applying; numbers and algebra; and shapes, space, and measures. Again, we calculated a composite math score, which was the mean of the three scales. Further information about NC teacher reports as used in TEDS can be found elsewhere (Haworth, Kovas, Petrill, & Plomin, 2007; Kovas et al., 2007; Walker, Petrill, Spinath, & Plomin, 2004;).

#### General cognitive ability

At age 7, we assessed the children’s general cognitive ability (g) using tests administered on the telephone. Our telephone adaptation of the tests retained the original test materials, and the administration procedure was closely aligned to the standard face-to-face procedure. Item lists were mailed to families in a sealed envelope prior to the test sessions. Two verbal and two nonverbal cognitive measures designed to yield an index of g were administered. The verbal measures were the Vocabulary and Similarities subtests of the Wechsler Intelligence Scale for Children-III-UK (Wechsler, 1992). The nonverbal measures were Picture Completion subtest from the Wechsler Scale and Conceptual Grouping from the McCarthy Scales of Children’s Abilities (McCarthy, 1972). Scores from our telephone adaptations of these standard cognitive tests have been shown to be substantially correlated with both subtest and composite scores from in-person assessments using the Stanford-Binet Intelligence Scale (Thorndike, Hagen, & Sattler, 1986) in 6- to 8-year-old children (Petrill, Rempell, Oliver, & Plomin, 2002).

We calculated a total g composite, which was the mean of the four subtests. In addition, we calculated Verbal and Nonverbal Composites as means of the two verbal and the two nonverbal subtests, respectively.

### Analyses

We used Pearson’s correlations to assess the relationship between these measures and the SNP-set scores. Before we conducted the analyses, we excluded those individuals who had major medical or perinatal problems, hearing difficulties, autism spectrum disorder, and English not the first spoken language. All measures were standardized on the basis of the whole sample to a mean of 0 and a standard deviation of 1 and were corrected for age and sex effects using a regression procedure.

## Results

We begin by presenting intercorrelations between our learning and cognitive measures and the original reading composite used by Meaburn et al. (in press) (see Table 1). The correlations are substantial for the NC English measures, especially for the NC reading measure and the NC English composite that include one of the measures included in the original composite used by Meaburn et al. Correlations between the original reading composite and our mathematics measures are also substantial (about .60). Correlations with g measures are lower—.41 for the Verbal Composite and .22 for the Nonverbal Composite.

**Table 1:** Correlations Between Original Reading Composite and Other Cognitive Measures

*Phenotype*	*Reading composite*^a^
NC English	
Speaking and listening	.632**
Reading	.937**
Writing	.691**
English composite	.859**
NC math	
Using and applying	.612**
Numbers and algebra	.630**
Shapes, space, and measures	.631**
Math composite	.665**
General cognitive ability	
Vocabulary	.386**
Similarities	.328**
Conceptual Grouping	.180**
Picture Completion	.156**
Verbal Composite	.410**
Nonverbal Composite	.224**
Composite g	.397**

*Note.*NC = National Curriculum.

a This reading composite is described in Meaburn et al. (in press) and is a composite score of the Twins Early Development Study NC reading measure and the Test of Word Reading Efficiency. This composite was used in the original association study that identified the 10 single nucleotide polymorphisms associated with reading ability.

** indicates significance at .01 alpha level.

The distribution for the SNP-set score is normal as shown in Figure 1 and in Meaburn et al. (in press). Table 2 shows the main results that test the generalist genes hypothesis: correlations between the reading SNP set and other literacy, math, and cognitive measures. Here we show correlations for those individuals who have data for all 10 SNPs and also correlations for individuals with missing data replaced with the population mean. Although the data with a missing data option contain less information, the sample size is much increased and provides greater power to detect small associations.

**Table 2:** Testing the Generalist Genes Hypothesis: Correlations Between Reading SNP-Set Scores and Other Measures of Literacy, Math, and Cognitive Abilities

*Phenotype*	*SNP set (complete data)^a^*	*SNP set (missing data option)^b^*
NC English		
Speaking and listening	*r*= .048 (*p*= .060), *N*= 1,538	*r*= .051** (*p*= .001), *N*= 3,924
Reading	*r*= .079** (*p*= .002), *N*= 1,536	*r*= .078** (*p*< .001), *N*= 3,919
Writing	*r*= .088** (*p*= .001), *N*= 1,529	*r*= .072** (*p*< .001), *N*= 3,903
English composite	*r*= .083** (*p*= .001), *N*= 1,543	*r*= .077** (*p*< .001) *N*= 3,935
NC math		
Using and applying	*r*= .109** (*p*< .001), *N*= 1,528	*r*= .072** (*p*< .001), *N*= 3,912
Numbers and algebra	*r*= .078** (*p*= .002), *N*= 1,526	*r*= .074** (*p*< .001), *N*= 3,909
Shapes, space, and measures	*r*= .086** (*p*= .001), *N*= 1,517	*r*= .071** (*p*< .001), *N*= 3,888
Math composite	*r*= .098** (*p*< .001), *N*= 1,533	*r*= .077** (*p*< .001), *N*= 3,923
General cognitive ability		
Vocabulary	*r*= .045 (*p*= .090), *N*= 1,442	*r*= .050** (*p*= .005), *N*= 3,158
Similarities	*r*= .050 (*p*= .058), *N*= 1,439	*r*= .042* (*p*= .017), *N*= 3,158
Conceptual Grouping	*r*= .035 (*p*= .179), *N*= 1,448	*r*= .026 (*p*= .151), *N*= 3,173
Picture Completion	*r*= .033 (*p*= .209), *N*= 1,445	*r*= .017 (*p*= .341), *N*= 3,172
Verbal Composite	*r*= .056* (*p*= .034), *N*= 1,434	*r*= .053** (*p*= .003), *N*= 3,145
Nonverbal Composite	*r*= .046 (*p*= .084), *N*= 1,445	*r*= .028 (*p*= .111), *N*= 3,169
Composite g	*r*= .066* (*p*= .012), *N*= 1,432	*r*= .052** (*p*= .003), *N*= 3,140

*Note.*NC = National Curriculum; SNPs = single nucleotide polymorphisms.

a These SNP-set analyses include only those individuals with data for all 10 SNPs.

b These SNP-set analyses include individuals with missing genotypes replaced with the population mean.

* indicates significance at .05 alpha level.

** indicates significance at .01 alpha level.

For individuals with complete data on all 10 SNPs, 9 of the 15 correlations between the cognitive measures and the SNP set were significant. Using the larger sample with a missing data option, this increased to 12 out of 15 SNPs. In these latter analyses, the only measures that were not significantly correlated with the SNP set were measures of the nonverbal component of *g*(Conceptual Grouping, Picture Completion, and the Nonverbal Composite).

## Discussion

The results provide strong support for the hypothesis of generalist genes that has until now largely depended on quantitative genetic analyses of twin data. A SNP set consisting of 10 SNPs identified on the basis of their association with individual differences in reading ability was significantly correlated with other literacy measures, components of mathematics performance, and general cognitive ability. Moreover, as predicted from multivariate genetic correlations, the reading SNP-set associations with these other literacy and learning ability measures were almost as strong as the association with reading itself. Also confirming multivariate genetic findings, the association with general cognitive ability was somewhat lower than for the other measures of literacy and mathematics. Delving further into general cognitive ability, an interesting and a reasonable result was that the associations with verbal tests (Vocabulary and Similarities) were greater than for nonverbal tests (Conceptual Grouping and Picture Completion).

It should be noted that the original reading measure used by Meaburn et al. was a general composite consisting of the TOWRE and a yearlong teacher assessment of reading based on UK NC criteria. The TOWRE is a brief test of word and nonword recognition, whereas the NC teacher assessment is a measure of many aspects of reading throughout the school year. Although these two measures are nearly as different as any two measures of reading could be, in line with the generalist genes hypothesis, the genetic correlation between them is .79 (Harlaar et al., 2005). The reason Meaburn et al. used such a general measure of reading in their genome-wide association study is that multivariate genetic research indicates that this is where the genetic action lies. That is, although multivariate genetic research also provides evidence for trait-specific genetic variance, most of the genetic variance for learning and cognitive abilities and disabilities is general, which is the essence of the generalist genes hypothesis. Nonetheless, it would be possible to use narrower measures of reading in an attempt to identify reading-specific genes.

The effect sizes of these associations are significant but small—as indicated in the introduction, association studies of complex traits and common disorders rarely find large effect sizes. In the original study reporting 10 SNPs associated with early reading, the average correlation of the associations was only .038; for this reason, the 10 SNPs were aggregated in a SNP set that correlated .105 with the reading composite (Meaburn et al., in press). In the present study, the reading SNP-set correlations with the other literacy, mathematics, and cognitive ability measures were generally lower than the SNP-set correlation with reading itself, but not much lower, as expected from the high genetic correlations in multivariate genetic analyses.

Although the SNP associations reported in the present study are significant despite their small effect sizes, our sample is the same sample used to identify the 10 SNP sets for reading (Meaburn et al., in press) and replication of these results in other samples is needed. Nonetheless, as they stand, these results provide an example of the usefulness of SNP sets even when the effect sizes of individual SNPs are very small. Bigger and better SNP sets are needed that account for as much as possible of the substantial heritability of learning abilities if SNP sets are to be useful in education to predict and prevent the development of disabilities. Educationally useful predictions of genetic risk could require hundreds or even thousands of SNPs, especially if SNPs associated with learning abilities and disabilities are identified not just at 7 years but at all ages, not just for generalist genes but also for specialist genes, and not just averaging across all environments but for specific interactions with family and school environments and with treatment and intervention programs. With microarrays, it would make little difference in terms of expense whether a learning abilities microarray had a hundred or a hundred thousand SNPs (Plomin & Schalkwyk, 2007). Moreover, in the not-too-distant future, it will be possible to sequence inexpensively all 3 billion nucleotide bases in each individual’s genome (Service, 2006), which would facilitate attempts to identify all DNA differences between people—not just SNPs but any type of polymorphism including structural variation in DNA such as copy number variants (Wong et al., 2007).

Although identifying such sets of genes associated with learning disabilities is unlikely to have direct impact on teachers in the classroom confronted with a particular child with a learning problem, the capacity to predict genetic risk from DNA will have far-reaching implications in terms of diagnosis, treatment, and prevention (Plomin & Walker, 2003). Gene-based diagnoses of learning disabilities are likely to be very different from current diagnoses. Most notably, the generalist genes hypothesis suggests that many of the same genes that predict reading disability will also predict math disability, although some genes will be specific to each disability. That is, a learning disabilities microarray in the future would mostly contain genes that can predict which children are likely to have general problems with reading and mathematics but it could also contain genes that can predict specific problems with reading or mathematics. Moreover, genes on a learning disabilities microarray that predict learning disabilities will also predict normal variation in learning abilities as well as high ability, which means that these genes will be useful for predicting the educational progress of all children, not just children at the low end of the normal distribution. Identifying these genes will lead to dimensional rather than diagnostic systems of classification of learning abilities and disabilities that are based on etiology rather than symptomatology. It will also lead to research on brain and mind pathways between genes and learning abilities and disabilities that can account for these general as well as specific effects (Kovas & Plomin, 2006).

A learning disabilities microarray could be even more important for treatment and prevention than for diagnosis. In terms of treatment, an untapped opportunity for genetic research is to identify genes that predict, not disorders themselves, but response to treatment. This goal is part of a “personalized medicine” movement toward individually tailored treatments rather than imposing one-size-fits-all treatments (Abrahams, Ginsburg, & Silver, 2005). It may be that education should follow the trend toward individualization by adopting specific learning plans for each child. Those children with special educational needs already have a certain level of personalized teaching plans (Department for Education and Skills, 2002). Further research into the environmental factors that are most relevant and their correlation or interaction with genetic effects could enlighten the options available for individualized learning for all children.

Identifying genes associated with learning disabilities will allow the prediction of learning problems very early in life. Rather than waiting until problems are so severe that they can no longer be ignored, finding genes will facilitate research on interventions that prevent learning disabilities from developing. The goal of early intervention fits with a general trend toward preventative medicine that is much more cost effective for children as well as for society. Interventions will rely on environmental engineering, such as teaching and classroom interventions, not on genetic engineering, which is not possible for complex traits that involve many genes of small effect size.

It could be argued that genetics is unimportant because we need to provide resources to prevent children from falling off the low end of the bell curve regardless of the causes of their poor performance. However, genetics is likely to facilitate the development of successful preventative interventions that can focus on diagnoses based on etiology rather than symptomatology. Genetics can also help to target children most likely to profit from interventions, which is important because successful prevention programs usually require extensive and intensive, and thus expensive, interventions (Hindson et al., 2005; Horowitz, 2004;).

What about the ethical issues raised by finding genes associated with learning abilities and disabilities? For example, will DNA microarrays justify social inequality? Knowledge alone does not account for societal and political decisions. Values are just as important in the decision-making process—decisions both good and bad can be made with or without knowledge. Finding genes that predict learning abilities and disabilities does not mean that we ought to put all our resources into educating the best readers and forgetting the rest. Depending on our values, genetics could be used to argue for devoting more resources to help disadvantaged children; genetics makes this view more palatable because it avoids assigning blame for poor reading solely to environmental failures of the school and family. The relationship between knowledge and values is a complicated area of philosophy, but surely, there is nothing to be gained by sticking our heads in the sand and pretending that genetic differences do not exist.
